# Surprisingly high number of Twintrons in vertebrates

**DOI:** 10.1186/1745-6150-8-4

**Published:** 2013-01-28

**Authors:** Jessin Janice, Marcin Jąkalski, Wojciech Makałowski

**Affiliations:** 1 Institute of Bioinformatics, Faculty of Medicine, University of Muenster, Niels Stensen Strasse 14, Muenster 48149, Germany

**Keywords:** Twintrons, Vertebrate genomes, Gene expression

## Abstract

Twintrons represent a special intronic arrangement in which introns of two different types occupy the same gene position. Consequently, alternative splicing of these introns requires two different spliceosomes competing for the same RNA molecule. So far, only two twintrons have been described in insects. Surprisingly, we discovered several such arrangements in vertebrate genomes, which are quite conserved throughout the lineages.

**Reviewers:**

This article was reviewed by Fyodor Kondrashow and Eugene Koonin.

## Findings

**Figure 1:**
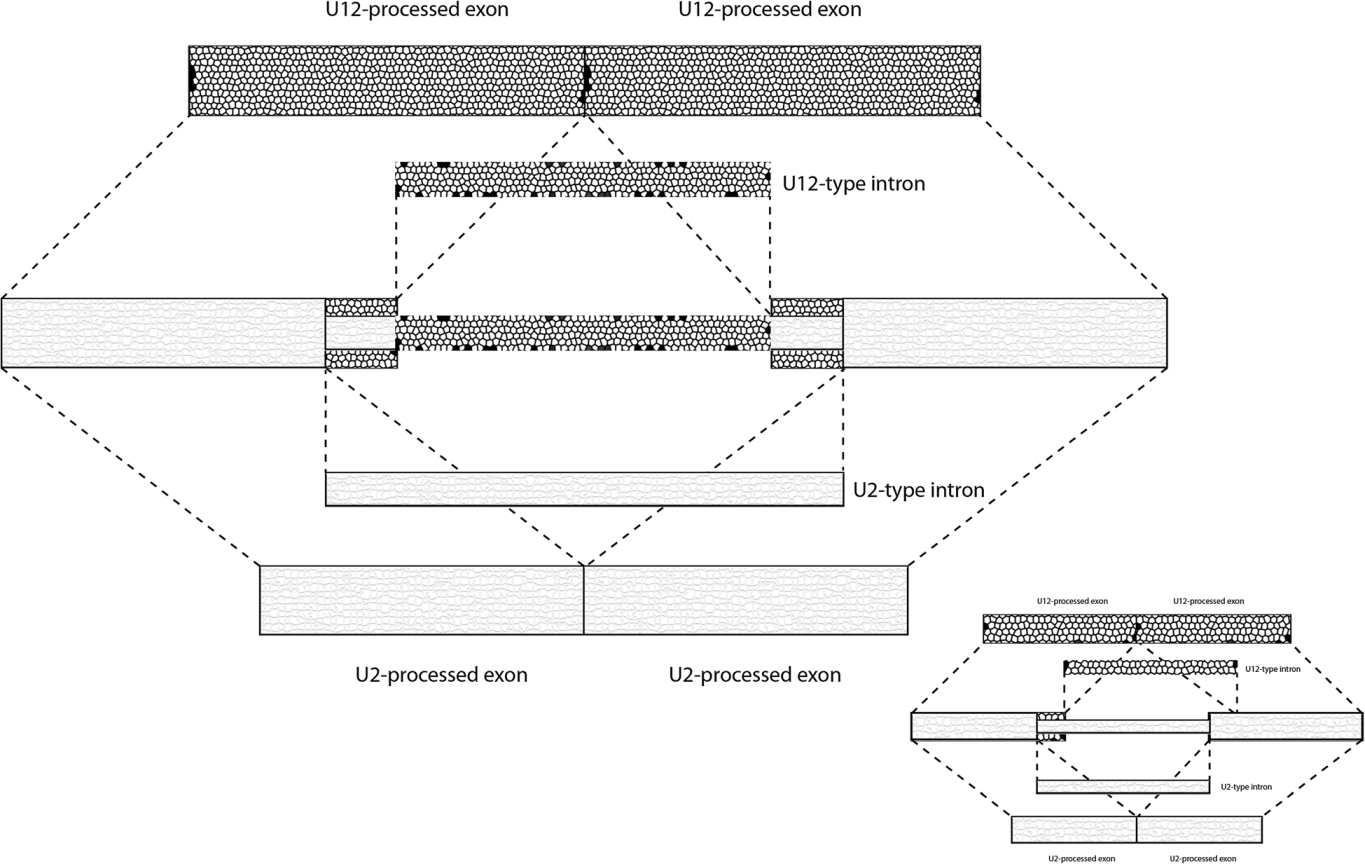
**Schematic representation of a twintron.** In most cases a set of splicing sites for a one type of spliceosome is nested in a set of splicing signals of another type of spliceosome (major cartoon). However, in a number of cases the splicing signals of the two spliceosomes are shifted, i.e. both 5^′^ and 3^′^ splicing signals of one spliceosome lay upstream of splicing singlas of another spliceosome (imbedded cartoon).

Most eukaryotic protein coding genes are interrupted by non-coding regions called introns [1], which are removed from pre-mRNA by a complex macromolecular machinery called spliceosome [2]. Interestingly, two types of spliceosomal introns exist that are processed by two distinct complexes. The major spliceosome recognizes and excises most of the introns (in humans, about 99.5% of the introns), while the rest are processed by the minor spliceosome. The two classes of introns are named after major RNA components of these spliceosomes: U2-type and U12-type introns, respectively [3]. Although the overall splicing mechanism of the two types of introns is very similar and the two spliceosomes share some components, it is believed that the two systems originated independently at different points in eukaryotic evolution [4]. It is intriguing that two types of introns can coexist in the same gene, which means that two large nucleoprotein complexes must operate simultaneously on a single pre-mRNA molecule. Even more surprising is the existence of so-called twintrons. We define twintrons as such an arrangement in which the alternatively spliced U12-type and U2-type introns occupy the same genomic location and are processed by different spliceosomes. Consequently, two spliceosomes must compete over the same RNA region to process a pre-mRNA (see Figure 1). This definition doesn’t imply any specific spatial relation of two types of introns, e.g. they don’t need to be nested one into another. In fact, one-third of the reported here twintrons are shifted, meaning that for instance both 5^′^ and 3^′^ splice sites of the U12-type intron lay upstream of U2-type splice sites (Figure 1 insert).

The spliceosomal twintron system was for the first time described in the gene *prospero* of *Drosophila melanogaster*[5]. The second intron of the gene contains two sets of splice sites (SS): a U12-type with an AT-AC termini and nested within a U2-type intron with a GT-AG termini resulting in a twenty-nine amino acids longer protein [6]. The U12-type intron of the *prospero* gene is an ancestral one, while the U2-type splice sites appeared early in the evolution of insects [7]. Recently, we have reported another insect-specific twintron in the *ZRSR2* gene. However, in this case, two sets of the splicing sites are not nested but shifted by several dozens of nucleotides and consequently, two protein isoforms are of a similar size [7]. Interestingly, the ancestral intron in this position was of the U2-type and the U12-type one is the first known case of *de novo* origination of a minor type intron. Nevertheless, we have hypothesized that the twintron arrangement is a safe pathway of intron type switching as it does not involve a dramatic change of spliceosomal specificity and allows step-wise evolutionary changes [7]. However, in both insect cases, twintron arrangement seems to be fixed and we did not observe type switching in either case. To further test our hypothesis, we expanded the search for twintrons into well-annotated vertebrate genomes.

A comprehensive scan of the human genome revealed eighteen twintronic arrangements within different genes (see Table 1). Interestingly, six of these twintrons consist of multiple alternative U2-type introns, with as many as seven U2-type intron variants in the *PRMT1* gene. Phylogenetic analysis revealed that for all these eighteen twintrons, the ancestral intron was of the U12-type and their presence is highly conserved throughout the vertebrate genomes (Additional file 1: Table S1). Two of the U12-type introns are even seen in the genome of *D. melanogaster*. We investigated several chordate genomes for twintron presence at the genomic and transcript levels. Surprisingly, comparative genomic analysis revealed a high evolutionary depth of the twintronic arrangements in vertebrates (Additional file 1:Table S1). In four cases, twintrons are apparent as far as in the lamprey genome, and in a few cases, they are also evident in amphibians*.*

**Table 1:** Details of the human genes harboring a twintron

**Gene**	**Function**	**Length of U12 variant (in aa)**	Length of U2 variant (in aa)*
*ACTR10*	This gene encodes actin involved in microtubule-based movement.	417	219 (U2-a), 219 (U2-b)
*C19orf54*	This gene encodes uncharacterized phosphoprotein.	351	139
*C1orf112*	Function unknown.	718	853 (U2-a), 606 (U2-b)
*C3orf17*	Function unknown.	567	392 (U2-a), 400 (U2-b)
*CTNNBL1*	Although the function of this protein has not been determined, the C-terminal portion of the protein has been shown to possess apoptosis-inducing activity.	563	376
*CUL4A*	This gene encodes ubiquitin ligase component of a multimeric complex involved in the degradation of DNA damage-response proteins.	789	149
*ESRP1*	This gene encodes RNA-binding protein that is an epithelial cell-type-specific splicing regulator.	742	206
*HNRPLL*	This gene encodes RNA-binding protein regulating activation-induced alternative splicing in T cells.	537	536
*NCBP2*	Component of the cap-binding complex (CBC), which binds to the monomethylated 5^′^ cap of nascent pre-mRNA in the nucleoplasm. The encoded protein has an RNP domain commonly found in RNA binding proteins, and contains the cap-binding activity. The CBC promotes pre-mRNA splicing, 3^′^-end processing, RNA nuclear export, and nonsense-mediated mRNA decay.	156	103
*PCID2*	This gene is expressed in immature and early-stage B lymphocytes and regulates expression of the mitotic checkpoint protein MAD2.	399	453 (U2-a), 292 (U2-b)
*PRMT1*	This gene encodes arginine methyltransferase that is responsible for the majority of cellular arginine methylation activity. Increased expression of this gene may play a role in many types of cancer.	NMD	371 (U2-a), 353 (U2-b), 346 (U2-c), 325 (U2-d), 213 (U2-e), 192 (U2-f)
*SLC9A7*	This gene encodes a sodium and potassium/ proton antiporter that is a member of the solute carrier family 9 protein family. It is primarily localized to the trans-Golgi network and is involved in maintaining pH homeostasis in organelles along the secretory and endocytic pathways.	725	727
*SPAG16*	This gene encodes protein kinase binding protein associated with the axoneme of sperm tail.	631	577
*SSR3*	This gene encodes the gamma subunit of glycosylated endoplasmic reticulum (ER) membrane receptor associated with protein translocation across the ER membrane.	198	174
*TAPT1*	This gene encodes a highly conserved, putative transmembrane protein. A mutation in the mouse ortholog of this gene results in homeotic, posterior-to-anterior transformations of the axial skeleton, which are similar to the phenotype of mouse homeobox C8 gene mutants.	567	338
*TTLL9*	This gene encodes a tubulin tyrosine ligase-like protein that forms polyglutamate side chains on tubulin.	347	439
*UBE2H*	This gene encodes a member of the ubiquitin-conjugating enzyme family. The modification of proteins with ubiquitin is an important cellular mechanism for targeting abnormal or short-lived proteins for degradation.	183	149
*ZNF207*	This gene encodes uncharacterized zing finger protein expressed in cultured breast cancer cells.	494	95 (U2-a), 74 (U2-b)

* In some cases, multiple U2-type introns are spliced from a single intronic position.

One of the interesting genes harboring a twintron is *PRMT1* (protein arginine N-methyltransferase 1), which functions as a histone methyltransferase specific for H4 [8]. There are more than twenty different splice variants reported for this gene. The second intronic position in most of the transcripts harbors a twintron where a U12-type intron is nested within a U2-type intron. This intronic region is excised in seven different ways, including an AT-AC U12-type intron. Although the 3^′^ SS is similar for all the introns except the U12-type intron, the 5^′^ SS varies extensively. The length of the introns also differs ranging from 209 nt for the shortest intron to 4,226 nt for the longest one. Interestingly, the U12-type intron belongs to the AT-AC type with an unusual AA terminus at the 3^′^ SS. Upon splicing, it produces a splice variant, which results in the Premature Termination Codon (PTC) and consequently is subjected to nonsense-mediated mRNA decay (NMD) in both the human and mouse genomes. Although the conserved motifs of minor intron are present in several vertebrate genomes, including opossum and *Xenopus*, we found solid evidence of the U12-type intron splicing only in humans and mice. Interestingly, in the platypus genome splicing signals for U12-type spliceosome has been muted and cannot be recognized by a minor spliceosome any longer. This may suggest that a twintron arrangement was a mediator of U12-type intron elimination from the host gene, in agreement to our original hypothesis [7].

To elucidate the role of alternative SSs in protein architecture, we scanned protein splice variants with InterProScan. Most of the protein isoforms of twintrons did not show any changes in the conserved motifs and structures, except for *HNRPLL* and *NCBP2*. Although the protein product of *HNRPLL* shows slight variations in the RNA recognition motif (RRM) for major and minor intron splice variants, the changes in the protein sequence and structure are insignificant (Additional file 2: Figure S1). The only gene that shows key structural variation is Nuclear Cap Binding Protein 2 (*NCBP2)*, which has RRM from the 42nd to the 112th amino acid of the U12-type splice variant (PDB ID – 3FEX) [9]. When the U2-type intron is spliced, a major portion of RRM is removed as a part of the U2-type intron (Additional file 3: Figure S2), most likely leading to a failure in binding with CAP80 to form the Cap-Binding Complex (CBC).

To comprehend the effect of twintrons in gene function and regulation, we looked at the expression patterns of all the twintronic protein isoforms. In many cases, the newly synthesized U2 splice variants are associated with cancerous tissues and in a few cases show tissue specific expression. This is especially evident in testicular tissue, as most of the newly evolved genes in testes seem to be preferentially expressed [10,11] (Additional file 4: Table S2). The U12-type splice variant of the gene *NCBP2* is expressed mainly in brain, thymus, uterus, lungs, testis, and several other tissues, whereas the U2-type splice variant is expressed mostly in tumors and cancerous tissues (Additional file 4: Table S2). *NCBP2* forms a heterodimer with CAP80 and plays a key role in the biogenesis of mRNAs, snRNAs, and microRNAs, and also in NMD. By a characterization of the U2 variant of *NCBP2,* Pabis et al. have discovered its physiological function in RNA processing [12]. U2 isoforms show precise subcellular distribution, associations with active transcription sites, and RNA processing proteins, showing several properties of RNA processing factors. Hence, the U2 splice variant may also play vital roles in RNA polymerase II transcription and/or co-transcriptional mRNA processing [12]. This gene serves as one of the best example of twintron regulation and utility. Only two spliceosomes twintrons have been reported previously [3,6,7]. Moreover, both are limited to insect genomes. Surprisingly, our scan of vertebrate genomes resulted in the discovery of several twintrons in higher animals. We expect that with the increase in transcriptomic and expression data, more twintrons will be found in the near future. As hypothesized previously, a twintron arrangement may serve as a safe pathway in intron type switching. Although we did not find clear evidence that this pathway was actually utilized, the *PRMT1* gene case may suggest that such a process happens in intron evolution. While there is a high chance of a splicing error in a gene with signals for both U2 and U12-type introns at the same position, the described twintrons are phylogeneticaly conserved, indicating their vital, yet elusive, role in the cell. Further analyses of the twintronic system should shed more light on the evolutionary importance of this fascinating phenomenon.

## Reviewers’ comments

### Reviewer 1 (Dr. Fyodor Kondrashov, Centre for Genomic Regulation, Spain)

This is a quaint study of the distribution of an interesting genomic element: nested introns where one of the introns is excised by the U-2 splicing system and the other by the U-12 system. It appears that over a dozen of such cases can be found throughout genes found in vertebrate genomes, some of them conserved throughout the clade.

I have two points and a question.

First, it is the definition of the term twintron. I am not a fan of this word, I think nested introns would have been a more descriptive term. Unfortunately for my sense of semantic taste I found that this terms is defined enough to appear in Wikipedia. It appears that the term was introduced in 1991 by authors that discovered nested group II introns in Euglena. Thus, according to the original (and Wikipedia) definition a twintron is any set of nested introns, belonging to the same splicing mechanism or not. This is at odds with the definition used by the authors and perhaps should be resolved. Perhaps a figure that demonstrates what a twintron looks like is called for: it would have been clearer to me what the authors mean.

Authors’ response: *Our definition of twintrons differs slightly from the original one and includes both nested and shifted arrangements. Although we provide a short twintron definition in the abstract, the full one is now provided in the body of the paper and accompanied by a figure*.

Second, the authors suggest that having such nested introns that are excised by two different spliceosomes can be an evolutionary mechanism of switching between the two intron types. However, perhaps this is at odds with the apparent conservation of such a setup - if this is a “safe pathway for intron switching” then certainly it does not appear to be a neutral one. Additionally, the mechanism that could turn an internal exon into an external one in a nested situation is not immediately clear to me.

Authors’ response: *We think that either of two introns can be switched off and this might be random process. We agree that presented examples don’t provide direct evidence that such a switch occurs. However, the fact that twintron arrangement is more common phenomena than anticipated provides indirect evidence that such a mechanism could be used during gene structure evolution*.

Is there a preference for U-2 or U-12 introns to be the external ones in the nested setup?

Authors’ response: *No, there’s no bias in the two types introns arrangement. In six cases U12-type intron is the internal one, while in four cases the arrangement is reversed. The rest of twintrons display shifted arrangement*.

### Reviewer 2 (Dr. Eugene Koonin, National Institutes of Health, USA)

This is quite an interesting short paper that reports a number of previously unnoticed twintrons and most importantly demonstrates their evolutionary conservation at considerable phylogenetic depths, with the implication of functional importance of the twintron structure itself. This is the major finding of the work, and it is certainly valuable. I am less enthusiastic of the two hypotheses proposed in the article, namely that twintrons could be an important intermediate along the path of elimination of U12 introns and that multiple protein isoforms produced by expression of twintron-containing genes might play a role in carcinogenesis. The first hypothesis, which is one of the main themes in the article, is of interest but I find the evidence quite limited. The authors might wish to expand the discussion. The idea about carcinogenesis, to me, is sheer, unwarranted speculation. The presence of additional splice variants in cancer samples might be caused by a variety of factors, above all the general deterioration of regulatory processes in tumors, and have nothing to do with carcinogenesis. I am not sure this is even worth a mention.

Authors’ response: *We agree that two hypotheses are highly speculative. As suggested, we have expanded the discussion of the first hypothesis and removed the second one from the manuscript*.

## Competing interests

The authors declare that they have no competing interests.

## Authors’ contribution

WM conceived and designed the project. JJ and MJ performed genomic analysis and JJ drafted the manuscript. All the authors contributed to the final version of the manuscript. All authors read and approved the final manuscript.

## Supplementary Material

Additional file 1: Table S1 Conservation of major and minor splice sites in vertebrates. Click here for file

Additional file 2: Figure S1 Superimposed 3D structures of U12 and U2-type splice variants of the gene *HNRPLL*. The structure is colored based on the secondary structure: red color for alpha-helices and yellow for beta-sheets. A black arrow indicates the variable amino acids. Click here for file

Additional file 3: Figure S2 Superimposed 3D structures of U12 and U2-type splice variants of the gene *NCBP2*. The 3D structure is colored based on the secondary structure: red color for alpha-helices and yellow for beta-sheets. RRM domain missing in the U2 splice variant is shown in blue. Click here for file

Additional file 4: Table S2 Expression data of splice variants of twintrons. Click here for file
