# Supplementary material for: Surprisingly high number of Twintrons in vertebrates
Source: Biol Direct. 2013 Jan 28;8:4. doi: 10.1186/1745-6150-8-4 (PMC3564746; doi:10.1186/1745-6150-8-4)
Supplement: Additional file 1: Table S1 — Conservation of major and minor splice sites in vertebrates. [file 1745-6150-8-4-S1.doc]

Table S1. Conservation of major and minor splice sites in vertebrates.

| ***Gene*** | ***Intron Type*** | ***Phylogeny (vertebrates)*** | | ***Confidence level [[1]](#footnote-2)*** | ***Drosophila’s Intron state*** |
| --- | --- | --- | --- | --- | --- |
| *ACTR10* | U12 | Human | Lamprey | 38/38 | Arp11 - Intron deletion |
|  | U2-a | Human | Lamprey | 25/29 |  |
|  | U2-b | Human | Lamprey | 31/40 |  |
| *C19orf54* | U12 | Human | Opossum | 26/40 | CG33108 – U12-type intron |
|  | U2 | Human | Zebrafish | 37/38 |  |
| *C1orf112* | U12 | Human | Stickleback | 32/37 | Absent |
|  | U2-a | Human | Orangutan | 3/19 |  |
|  | U2-b | Human | Marmoset | 4/19 |  |
| *C3orf17* | U12 | Human | Lizard | 31/34 | Absent |
|  | U2-a | Human | Sloth | 31/34 |  |
|  | U2-b | Human | Sloth | 31/34 |  |
| *CTNNBL1* | U12 | Human | Lamprey | 41/41 | CG11964 – Intron deletion |
|  | U2 | Human | Elephant | 10/35 |  |
| *CUL4A* | U12 | Human | Lamprey | 32/32 | Cul-4 – Intron deletion |
|  | U2 | Human | Chicken | 30/37 |  |
| *ESRP1* | U12 | Human | Xenopus | 35/36 | Glo – Intron deletion |
|  | U2 | Human | Tarsier | 3/6 |  |
| *HNRPLL* | U12 | Human | Lamprey | 34/34 | Sm - Intron deletion |
|  | U2 | Human | Sloth | 21/34 |  |
| *NCBP2* | U12 | Human | Lamprey | 33/34 | Cpb20 - short protien |
|  | U2 | Human | Lamprey | 34/40 |  |
| *PCID2* | U12 | Human | Lamprey | 34/35 | PCID2 - Intron less gene |
|  | U2-a | Human | Lamprey | 23/35 |  |
|  | U2-b | Human | Elephant | 20/23 |  |
| *PRMT1* | U12 | Human | Xenopus | 21/22 | Art1 - Intron Deletion |
|  | U2-a | Human | Xenopus | 23/23 |  |
|  | U2-b | Human | Oppasum | 21/29 |  |
|  | U2-c | Human | Platypus | 26/29 |  |
|  | U2-d | Human | Microbat | 17/21 |  |
|  | U2-e | Human | Armadillo | 23/32 |  |
|  | U2-f | Human | Dog | 17/20 |  |
| *SLC9A7* | U12 | Human | Lamprey | 34/34 | Nhe3 - Intron Deletion |
|  | U2 | Human | Oppasum | 20/34 |  |
| *SPAG16* | U12 | Human | Sloth | 27/28 | Absent |
|  | U2 | Human | Megabat | 14/26 |  |
| *SSR3* | U12 | Human | Lamprey | 33/37 | CG5885 - intron deletion |
|  | U2 | Human | Lamprey | 13/27 |  |
| *TAPT1* | U12 | Human | Zebra fish | 42/42 | CG7218 - Intron deletion |
|  | U2 | Human | Zebra fish | 41/41 |  |
| *TTLL9* | U12 | Human | Chicken | 31/35 | Absent |
|  | U2 | Human | Baboon | 5/21 |  |
| *UBE2H* | U12 | Human | Elephant | 19/19 | Ubc-E2H – Intronless gene |
|  | U2 | Human | Elephant | 17/21 |  |
| *ZNF207* | U12 | Human | Zebrafish | 28/40 | CG17912 – U12-type intron |
|  | U2-a | Human | Marmoset | 7/31 |  |
|  | U2-b | Human | Zebrafinch | 31/31 |  |

Confidence level is the scale what we used here to confirm the occurrence of twintrons in other vertebrates. Number in the numerator refers to the number of vertebrates possessing this splice site and the number in denominator refers to the number of total vertebrate genomic sequence available for the study. Since not all the genomic regions of all the vertebrates are sequenced, there are discrepancies in the number of genomic regions available.

1. [↑](#footnote-ref-2)
