# Supplementary material for: Surprisingly high number of Twintrons in vertebrates
Source: Biol Direct. 2013 Jan 28;8:4. doi: 10.1186/1745-6150-8-4 (PMC3564746; doi:10.1186/1745-6150-8-4)
Supplement: Additional file 2: Figure S1 — Superimposed 3D structures of U12 and U2-type splice variants of the gene HNRPLL. The structure is colored based on the secondary structure: red color for alpha-helices and yellow for beta-sheets. A black arrow indicates the variable amino acids. [file 1745-6150-8-4-S2.doc]

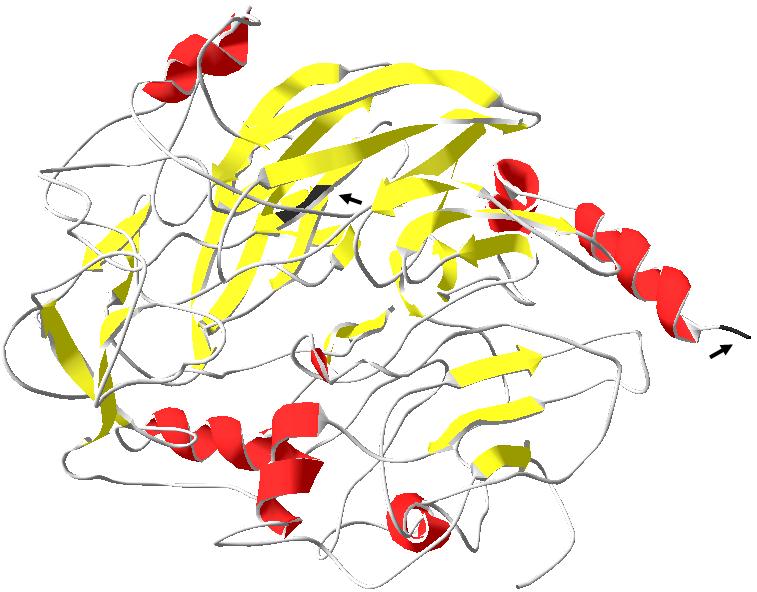


Figure S1. Superimposed 3D structures of U12 and U2-type splice variants of the gene HNRPLL. The structure is colored based on the secondary structure, red color for alpha-helices and yellow for beta-sheets. A black arrow indicates the variable amino acids.
