# Supplementary material for: Surprisingly high number of Twintrons in vertebrates
Source: Biol Direct. 2013 Jan 28;8:4. doi: 10.1186/1745-6150-8-4 (PMC3564746; doi:10.1186/1745-6150-8-4)
Supplement: Additional file 3: Figure S2 — Superimposed 3D structures of U12 and U2-type splice variants of the gene NCBP2. The 3D structure is colored based on the secondary structure: red color for alpha-helices and yellow for beta-sheets. RRM domain missing in the U2 splice variant is shown in blue. [file 1745-6150-8-4-S3.doc]

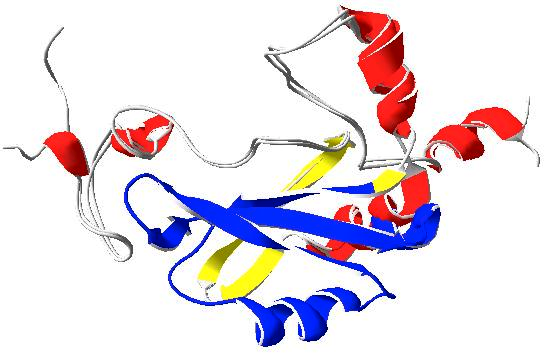


Figure S2. Superimposed 3D structures of U12 and U2-type splice variants of the gene *NCBP2*. The 3D structure is colored based on the secondary structure: red color for alpha-helices and yellow for beta-sheets. RRM domain missing in the U2 splice variant is shown in blue.
