# Supplementary material for: Surprisingly high number of Twintrons in vertebrates
Source: Biol Direct. 2013 Jan 28;8:4. doi: 10.1186/1745-6150-8-4 (PMC3564746; doi:10.1186/1745-6150-8-4)
Supplement: Additional file 4: Table S2 — Expression data of splice variants of twintrons. [file 1745-6150-8-4-S4.doc]

Table S2. Expression data of splice variants of twintrons.

| **Gene** | **Function** | **Length of U12 variant (in aa)** | **Length of U2 variant (in aa)*** |
| --- | --- | --- | --- |
| *ACTR10* | 417 | U12 | brain (21), placenta cot 25-normalized (21), placenta (9), liver (7), lung (6), eye (5), fetal brain (5), adult brain (4), ovarian tumor (4), ovary (4), pooled brain, lung, testis (4), testis (4), total brain (4), cerebellum (3), chondrosarcoma (3), dorsal root ganglia (3), hypothalamus (3), left pubic bone (3), neuroblastoma cot 50-normalized (3), uterus (3), B cells from burkitt lymphoma (2), embryonic stem (2), embryonic stem cell, retinoic acid andmitogen-treated hes cell line H7 (2), heart (2), hippocampus (2), lens (2), pancreas (2), parathyroid gland (2), parathyroid tumor (2), primary lung cystic fibrosis epithelialcells (2), primary lung epithelial cells (2), purified pancreatic islet (2), retina (2), skin (2), subthalamic nucleus (2), T cells (jurkat cell line) (2) and 30 other tissues |
|  | 219 | U2-a | placenta cot 25-normalized (8), lung (3), T cells (jurkat cell line) cot10-normalized (3), adult brain (2) and 9 other tissues |
|  | 219 | U2-b | purified pancreatic islet (2) and placenta cot 25-normalized |
| *C19orf54* | 351 | U12 | eye (9), retina (7), brain (3), astrocytoma grade IV, cell line (2) and 36 other tissues |
|  | 139 | U2 | lung (1) and also predicted NMD candidate |
| *C1orf112* | 718 | U12 | neuroblastoma cot 25-normalized (2), caudate nucleus (1), cervical carcinoma cell line (1), cervix (1) and 5 other tissues |
|  | 853 | U2-a | hypothalamus (5), parathyroid gland (3), parathyroid tumor (3), testis (3) and 17 other tissues |
|  | 606 | U2-b | caudate nucleus (1), testis (1), thymus (1) |
| *C3orf17* | 567 | U12 | brain (13), placenta (5), thymus (5), testis (4), uterus (4), bladder (3), hippocampus (3), spleen (3), trachea (3), fetal brain (2), liver (2), neuroblastoma (2), stomach (2), T cells from T cell leukemia (2), and 16 other tissues |
|  | 400 | U2-a | brain (2), substantia nigra (2), caudate nucleus (1), cerebellum (1) and 3 other tissues |
|  | 392 | U2-b | thymus (1) |
| *CTNNBL1* | 563 | U12 | lung (21), placenta (20), liver (18), epidermoid carcinoma, cell line (15), hepatocellular carcinoma, cell line (14), lymph (14), lymphoma, cell line (14), skin (12), choriocarcinoma (11), brain (7), salivary gland (7), breast carcinoma (6), eye (6), ovary (6), pancreas (6), prostate (6), testis (6), thymus (6), uterus (6), amelanotic melanoma, cell line (5), kidney (5), cerebellum (4), large cell carcinoma (4), spleen (4), stomach (4), adenocarcinoma cell line (3), ascites (3), carcinoma, cell line (3), cervix (3), colon (3), ductal carcinoma, cell line (3), heart (3), melanotic melanoma (3), melanotic melanoma, high MDR (cell line) (3), normal nasopharynx (3), alzheimer cortex (2), b-cells (2), breast, tumor tissue (2), carcinoid (2), embryonic stem cell, retinoic acid andmitogen-treated hes cell line H7 (2), esophageal, tumor tissue (2), fetal eyes (2), neuroblastoma, cell line (2), nose (2), olfactory epithelium (2), ovarian tumor (2), primary b-cells from tonsils (cell line) (2), retinoblastoma (2), small intestine (2), synovial membrane tissue from rheumatioidarthritis (2), thalamus (2), uterine (2), and 48 other tissues |
|  | 376 | U2 | testis (1) |
| *CUL4A* | 789 | U12 | brain (11), testis (9), hippocampus (4), cerebellum (3), lung (3), trachea (3), astrocytoma grade IV, cell line (2), colon (2), ductal carcinoma, cell line (2), epidermoid carcinoma, cell line (2), human embryonic stem cells differentiated toan early endodermal cell type (2), juvenile granulosa tumor (2), neuroblastoma cells (2), neuroblastoma cot 25-normalized (2), pancreas (2), and 16 other tissues |
|  | 149 | U2 | testis and tounge |
| *ESRP1* | 742 | U12 | placenta cot 25-normalized (2), bladder (1), breast, adenocarcinoma (1), colon (1) and 5 other tissues |
|  | 206 | U2 | colon (1), colon tumor RER+ (1), embryonic stem cells, dmso-treated H9 cellline (1), skin (1) and 1 other tissue |
| *HNRPLL* | 537 | U12 | brain (19), testis (14), breast carcinoma (9), hypothalamus (9), uterus (9), eye (8), kidney (8), lung (7), pancreas (7), pooled germ cell tumors (7), stomach (7), glioblastoma (pooled) (6), 2 pooled tumors (clear cell type) (5), bladder (5), cervix (5), colon (5), islets of langerhans (4), breast (3), carcinoma, cell line (3), embryonic stem cell, retinoic acid andmitogen-treated hes cell line H7 (3), embryonic stem cells, cell lines H1, H7, andh9 (3), hippocampus (3), liver (3), placenta (3), serous papillary carcinoma, high grade, 2pooled tumors (3), skin (3), whole brain (3), amygdala (2), ascites (2), blood vessels - aorta, basilar and artery (2), chondrosarcoma (2), cochlea (2), ear (2), embryonic stem cells, dmso-treated H9 cellline (2), fetal eye (2), glioblastoma (2), lymph node (2), lymphoma, follicular mixed small and largecell (2), placenta cot 25-normalized (2), pooled human melanocyte, fetal heart, andpregnant uterus (2), poorly differentiated adenocarcinoma withsignet ring cell features (2), prostate (2), retinoblastoma (2), senescent fibroblast (2), synovial membrane tissue from rheumatioidarthritis (2), transitional cell papilloma, cell line (2), uterus tumor (2) and 45 other tissues |
|  | 536 | U2 | leiomyosarcoma (1), placenta cot 25-normalized (1), uterus (1), whole brain (1) and 1 other tissue |
| *NCBP2* | 156 | U12 | brain (19), uterus (8), coronary artery (6), skin (6), lung (5), pancreas (5), placenta (5), thymus (5), eye (4), insulinoma (4), kidney (4), leiomyosarcoma (4), melanotic melanoma (4), placenta cot 25-normalized (4), testis (4), thalamus (4), amygdala (3), blastocyst (3), breast (3), cerebellum (3), cervix (3), liver (3), mammary adenocarcinoma, cell line (3), pluripotent cell line derived fromblastocyst inner cell mass (3), prostate (3), retinoblastoma (3), small intestine (3), spleen (3), substantia nigra (3), caudate nucleus (2), embryonic stem cell, retinoic acid andmitogen-treated hes cell line H7 (2), embryonic stem cells, dmso-treated H9 cellline (2), fetal brain (2), fetal liver (2), hepatoblastoma (2), human embryonic stem cells differentiated toan early endodermal cell type (2), neuroblastoma (2), rectum (2), subthalamic nucleus (2) and 19 other tissues |
|  | 103 | U2 | uterus (18), lung (12), brain (11), eye (11), breast carcinoma (10), stomach (7), colon (6), heart (6), skin (6), testis (6), glioblastoma (pooled) (5), prostate (5), breast (4), fetal eye (4), fibrosarcoma (4), hippocampus (4), ovary (4), placenta normal (4), bladder (3), bone (3), carcinoid (3), chondrosarcoma (3), cochlea (3), ear (3), embryonal carcinoma, cell line (3), embryonic stem cells, cell lines H1, H7, andh9 (3), embryonic stem cells, embryoid bodiesderived from H1, H7 and H9 cells (3), germinal center B cell (3), head neck (3), kidney (3), leiomyosarcoma (3), liver (3), melanotic melanoma (3), ovarian tumor (3), pancreas (3), poorly differentiated adenocarcinoma withsignet ring cell features (3), primary lung epithelial cells (3), retinoblastoma (3), serous papillary carcinoma, high grade, 2pooled tumors (3), adenocarcinoma (2), adrenal cortex carcinoma, cell line (2), adrenal gland (2), anaplastic oligodendroglioma (2), bone marrow stroma (2), colon ins (2), embryonic stem cell, retinoic acid andmitogen-treated hes cell line H7 (2), fetal eyes, lens, eye anterior segment,optic nerve, retina, retina foveal and macular, RPE andchoroid (2), insulinoma (2), kidney tumor (2), left pelvis (2), lung tumor (2), marrow (2), moderately-differentiated adenocarcinoma (2), multiple sclerosis lesions (2), placenta (2), pooled germ cell tumors (2), posterior rhombomeres 5-8 (4 pooled) (2), senescent fibroblast (2), squamous cell carcinoma (2), transitional cell papilloma, cell line (2), whole embryo, mainly head (2) and 2 pooled wilms' tumors, and 64 other tissues |
| *PCID2* | 399 | U12 | testis (4), brain (3), ovary, tumor tissue (3), germinal center B cell (2), liver (2), neuroblastoma cells (2), neuroblastoma cot 25-normalized (2), pancreas (2), purified pancreatic islet (2), thymus (2) and 8 other tissues*. |
|  | 453 | U2-a | placenta (2), endometrium (1) |
|  | 292 | U2-b | fetal brain (1), skeletal muscle (1) |
| *PRMT1* | N/A | U12 | Brain, NMD |
|  | 371 | U2-a | eye (7), brain (4), lymph (3), lymphoma, cell line (3), retina (3), adenocarcinoma (2), adenocarcinoma, cell line (2), CNCAP(3)T-225 cell line (2), human retina (2), liver (2), lung (2), neuroblastoma cells (2), neuroblastoma cot 50-normalized (2), ovarian tumor (2), ovary (2), pancreas (2), prostate (2), retinoblastoma (2), t-lymphocytes (2) and 18 other tissues. |
|  | 353 | U2-b | brain (17), lung (15), uterus (13), eye (12), placenta cot 25-normalized (10), embryonic stem (9), leiomyosarcoma (8), skin (7), neuroblastoma cot 25-normalized (6), prostate (6), ascites (5), B cells from burkitt lymphoma (5), embryonal carcinoma, cell line (5), stomach (5), testis (5), adenocarcinoma, cell line (4), bone (4), embryonic stem cell, retinoic acid andmitogen-treated hes cell line H7 (4), epidermoid carcinoma, cell line (4), lymph (4), melanotic melanoma (4), retinoblastoma (4), breast (3), carcinoma, cell line (3), cervix (3), human embryonic stem cells (3), melanotic melanoma, cell line (3), ovary (3), small cell carcinoma (3), anaplastic oligodendroglioma with 1p/19qloss (2), B cells (ramos cell line) (2), B cells (ramos cell line) cot 25-normalized (2), blastocyst (2), burkitt lymphoma (2), chondrosarcoma grade II (2), embryonic stem cells, dmso-treated H9 cellline (2), enchondroma cell line (2), heart (2), human embryonic stem cells differentiated toan early endodermal cell type (2), left pelvis (2), liver (2), lymphoma, cell line (2), medulla (2), neuroblastoma cells (2), neuroblastoma, cell line (2), optic nerve (2), ovarian tumor (2), pancreas (2), placenta (2), pluripotent cell line derived fromblastocyst inner cell mass (2), primary lung epithelial cells (2), RPE and choroid (2), senescent fibroblast (2), spleen (2), subchondral bone (2), T cells (jurkat cell line) cot10-normalized (2), and 40 other tissues |
|  | 346 | U2-c | data unknown |
|  | 325 | U2-d | skin (16), stomach (11), ascites (9), head neck (8), prostate (8), brain (7), amelanotic melanoma, cell line (5), breast (5), carcinoma, cell line (5), melanotic melanoma (5), uterus (5), adenocarcinoma, cell line (4), embryonic stem cells, cell lines H1, H7, andh9 (4), heart (4), liver (4), ovary (4), adenocarcinoma cell line (3), colon (3), embryonic stem cells, dmso-treated H9 cellline (3), lung (3), lymph (3), pancreas (3), placenta (3), squamous cell carcinoma (3), t-lymphocytes (3), bladder (2), cervix (2), embryonic stem cells, embryoid bodiesderived from H1, H7 and H9 cells (2), epidermis (2), kidney (2), leukocyte (2), lymphoma, cell line (2), neuroblastoma (2), purified pancreatic islet (2), small cell carcinoma (2), thyroid (2), transitional cell papilloma, cell line (2) and 34 other tissues |
|  | 213 | U2-e | eye, retinoblastoma |
|  | 192 | U2-f | eye |
| *SLC9A7* | 725 | U12 | B cell (7), senescent fibroblast (3), tongue (3) and 13 other tissues |
|  | 727 | U2 | N/A |
| *SPAG16* | 631 | U12 | testis (9), brain (2), hypothalamus (2), myeloma (2) and 13 other tissues |
|  | 577 | U2 | breast carcinoma (3), hippocampus (3), insulinoma (2), pancreas (2) and 7 other tissues |
| *SSR3* | 198 | U12 | colon (1), colonic mucosa from 3 patients with crohn'sdisease (1), corpus callosum (1), trachea (2), brain (1), glioblastoma (1) |
|  | 174 | U2 | brain (1), liver and spleen (1), primitive neuroectoderm (1) |
| *TAPT1* | 567 | U12 | testis (14), kidney (8), lung (8), brain (7), eye (7), germinal center B cell (7), pancreas (7), placenta (7), breast carcinoma (6), colon (6), carcinoid (5), uterus (5), chondrosarcoma (4), left pelvis (4), liver and spleen (4), prostate (4), retina (4), 2 pooled tumors (clear cell type) (3), embryonal carcinoma, cell line (3), marrow (3), pooled human melanocyte, fetal heart, andpregnant uterus (3), thymus (3), whole brain (3), alzheimer cortex (2), chondrosarcoma grade II (2), epithelioid carcinoma (2), human embryonic stem cells (2), hypothalamus (2), insulinoma (2), islets of langerhans (2), melanocyte (2), pancreatic islet (2), poorly-differentiated endometrialadenocarcinoma, 2 pooled tumors (2), retina foveal and macular (2), trachea (2), whole embryo, mainly head (2), adrenal cortex carcinoma, and 39 other tissues |
|  | 338 | U2 | thymus (1) |
| *TTLL9* | 347 | U12 | breast (8), eye (8), fetal eye (6), mammary adenocarcinoma, cell line (6), placenta (5), bone (4), brain (4), lung (4), melanocyte (4), bladder (3), carcinoma, cell line (3), liver (3), osteosarcoma, cell line (3), ovary (3), pancreas (3), prostate (3), purified pancreatic islet (3), uterus (3), bone marrow (2), cell lines (2), chondrosarcoma (2), CNCAP(3)T-225 cell line (2), kidney (2), liver and spleen (2), ovarian tumor (2), pooled colon, kidney, stomach (2), skin (2), testis (2) and 38 other tissues |
|  | 439 | U2 | germinal center B cell (2), kidney (1) |
| *UBE2H* | 183 | U12 | lung (7), brain (4), breast carcinoma (4), placenta (4), pooled human melanocyte, fetal heart, andpregnant uterus (4), squamous cell carcinoma, poorlydifferentiated (4 pooled tumors, including primary andmetastatic) (4), melanocyte (3), pre-eclamptic placenta (3), frontal lobe (2), liver and spleen (2), pancreas (2), prostate (2), purified pancreatic islet (2), senescent fibroblast (2), uterus (2), and 21 other tissues |
|  | 149 | U2 | liver, tumor tissue |
| *ZNF207* | 494 | U12 | leiomyosarcoma (5), uterus (5), brain (4), melanotic melanoma (3) and 29 other tissues |
|  | 95 | U2-a | N/A |
|  | 74 | U2-b | testis |

*Other tissues – the tissues in which the tracsript are expressed only once.
